# Health Information Exchange Implementation: Lessons Learned and Critical Success Factors From a Case Study

**DOI:** 10.2196/medinform.3455

**Published:** 2014-08-15

**Authors:** Sue S Feldman, Benjamin L Schooley, Grishma P Bhavsar

**Affiliations:** ^1^Central Virginia Health NetworkRichmond, VAUnited States; ^2^Arnold School of Public HealthUniversity of South CarolinaColumbia, SCUnited States; ^3^School of Information Systems and TechnologyClaremont Graduate UniversityClaremont, CAUnited States; ^4^Department of Integrated Information TechnologyUniversity of South CarolinaColumbia, SCUnited States

**Keywords:** health information exchange, blended value collaboration enactment framework, HIE value proposition, interorganizational systems, HIE implementation

## Abstract

**Background:**

Much attention has been given to the proposition that the exchange of health information as an act, and health information exchange (HIE), as an entity, are critical components of a framework for health care change, yet little has been studied to understand the value proposition of implementing HIE with a statewide HIE. Such an organization facilitates the exchange of health information across disparate systems, thus following patients as they move across different care settings and encounters, whether or not they share an organizational affiliation. A sociotechnical systems approach and an interorganizational systems framework were used to examine implementation of a health system electronic medical record (EMR) system onto a statewide HIE, under a cooperative agreement with the Office of the National Coordinator for Health Information Technology, and its collaborating organizations.

**Objective:**

The objective of the study was to focus on the implementation of a health system onto a statewide HIE; provide insight into the technical, organizational, and governance aspects of a large private health system and the Virginia statewide HIE (organizations with the shared goal of exchanging health information); and to understand the organizational motivations and value propositions apparent during HIE implementation.

**Methods:**

We used a formative evaluation methodology to investigate the first implementation of a health system onto the statewide HIE. Qualitative methods (direct observation, 36 hours), informal information gathering, semistructured interviews (N=12), and document analysis were used to gather data between August 12, 2012 and June 24, 2013. Derived from sociotechnical concepts, a Blended Value Collaboration Enactment Framework guided the data gathering and analysis to understand organizational stakeholders’ perspectives across technical, organizational, and governance dimensions.

**Results:**

Several challenges, successes, and lessons learned during the implementation of a health system to the statewide HIE were found. The most significant perceived success was accomplishing the implementation, although many interviewees also underscored the value of a project champion with decision-making power. In terms of lessons learned, social reasons were found to be very significant motivators for early implementation, frequently outweighing economic motivations. It was clear that understanding the guides early in the project would have mitigated some of the challenges that emerged, and early communication with the electronic health record vendor so that they have a solid understanding of the undertaking was critical. An HIE implementations evaluation framework was found to be useful for assessing challenges, motivations, value propositions for participating, and success factors to consider for future implementations.

**Conclusions:**

This case study illuminates five critical success factors for implementation of a health system onto a statewide HIE. This study also reveals that organizations have varied motivations and value proposition perceptions for engaging in the exchange of health information, few of which, at the early stages, are economically driven.

## Introduction

### Investing in Health Information Technology

The Health Information Technology for Economic and Clinical Health, HITECH, Act, enacted as part of the American Recovery and Reinvestment Act of 2009, set a national goal of investing in health information technology to improve health care delivery. To meet this goal, the electronic exchange of health information between providers is essential to ensure coordinated, efficient, and quality care. This exchange can be accomplished through various local, regional, or statewide organizations that build the infrastructure to facilitate secure health information exchange (HIE); the federal government has already entered into cooperative agreements with 56 states and territories to fund infrastructure to enable these efforts [1]. HIE is a complex and emergent system of structures and actions, which varies in scope and scale. The current study discusses HIE as both the *act* of exchanging health information between two collaborating organizations, and the *entity* that facilitates such exchange. The goal of this study, which focuses on the implementation of HIE as an interorganizational health care system, is to understand the organizational motivations and value propositions apparent during HIE implementation. These value propositions are analyzed through an interorganizational system (IOS) information technology (IT) governance lens that considers the technical, organizational, and governance dimensions of HIE value. We apply the framework to: (1) evaluate HIE implementation challenges, successes, and lessons learned; and (2) extract value propositions across organizational stakeholders.

### Health Information Exchange

Much attention has been given to the proposition that the exchange of health information is a critical component of a framework for health care change, the Triple Aim being: (1) better patient experiences through quality and satisfaction; (2) better health outcomes of populations; and (3) reduction of per capita cost of health care [2]. These changes will rely on organizational entities that have entered cooperative agreements with the federal government to provide technical infrastructure, organizational structure, and governance mechanisms for completing the act of HIE [1]. The act of HIE, described various ways in the literature, can be conducted across affiliated physicians’ offices, hospitals, and clinics; or can occur between completely disparate systems [3,4]. HIE across disparate systems allows clinical information to follow patients as they move across different care settings, whether or not they share an organizational affiliation. For example, this might include a hospital or health system connected to an HIE network that is, in turn, connected to several for-profit and not-for-profit competing hospitals or health systems networks. On a broad scale, this type of HIE holds great promise for achieving the Triple Aim goals.

### Health Information Exchange Benefits and Challenges

The implementation and use of HIE technology have influenced patient care by allowing providers direct access to health information, reducing time to obtain health information, and increasing providers’ awareness of patient interactions with the health care system [5]. The benefits and challenges of HIE have been studied in prior research. Regarding patient experiences, previous studies have found improved coordination of care and enhanced patient health outcomes for human immunodeficiency virus patients [6], higher patient satisfaction [7], informed patient care [8], efficient care [8], and positive patient perception of impact on care coordination [9]. However, others have found that the benefits of HIE in relation to patient outcomes are limited [10].

From a broader provider and patient perspective, timely sharing of a patient’s clinical information can improve the accuracy of diagnoses, reduce the number of duplicative tests, prevent hospital readmissions, and prevent medication errors [3,11,12]. From a public health perspective, the exchange of health information has fostered positive relationships with public health agencies [13], improved public health surveillance [14], and increased the efficiency and quality of public health reporting [15].

Though the theoretical case for HIE on reducing the utilization and cost of health care services is compelling and has received a great deal of emphasis [16], empirical evidence is still inconclusive [17,18]. This may reflect the nascent nature of HIE, especially between disparate systems, and the fact that these systems and the context are complex and emergent. For example, HIE has faced challenges like those of other new IT initiatives, disparate and noninteroperable information technologies [19,20]; a range of technical, work flow, and organizational challenges to exchanging information [17,21]; and a variety of governance challenges [22,23]. Yet the HIEs that have continued to operate have done so with evolving and maturing technical, organizational, and governance structures [24-27].

Still, much more research is needed to understand HIE, how it operates, what factors contribute to success, and even how success should be defined. At this early phase of HIE development and implementation, it is important to study the system and its context to improve upon existing methods, tools, and frameworks. This study investigates the value of HIE from an IT implementation perspective. Specifically, it asks what motivations, challenges, and successes lead to value realization across organizations working together to on-board to a state HIE? The sociotechnical systems (STS) approach of this study applies an IOS framework developed through previous work to understand blended value across participating organizations.

### Sociotechnical Systems Approach

An STS approach examines social/community links to the technical [28]. STS design includes several levels of abstraction including mechanical (hardware), informational (software), psychological (person), and social (community). Such an inclusive approach is aimed at understanding interdependent linkages between increasingly complex social and technological components. Working together, these components consider social motivations and accomplish a set of social goals that otherwise would not be realized.

The highest-order social benefit (human life) of health information sharing are stated quite succinctly by Porter and Teisburg [29], “The social benefits of results information will be even greater in health care than in the financial markets, because the physical well being of Americans is at stake.” Social value factors include the range of intangible and actor-based or organizational considerations that contribute to collaboration success. Furthermore, two studies almost 20 years apart suggest that successful IT project advancement is frequently associated with social elements [30,31]. In his book on infrastructure delivery in public-private collaborations, Mody [32] draws from the railway and transportation systems examples to suggest that social considerations, such as being able to deliver goods and information to the right place at the right time, might exceed those of economic returns and could exert greater significant pressure.

Therefore, examination of the social motivations and benefits deserve to be considered in a different light than a customary return on investment model commonly considered in information exchange in the business world. This blended value proposition has been defined as the combination of social and economic value used to maximize total returns where “the core nature of investment and return is not a tradeoff between social and financial interest, but rather the pursuit of an embedded value proposition composed of both” [33]. Emerson [33] continues,

Societies cannot function strictly on the basis of their economic enterprise. It is social commerce that allows individuals and institutions to pursue the traditional financial returns sought by mainstream financial capital market players.

J Emerson

An STS approach, which focuses on systems that are both technologically sound and socially sustainable [34], has been applied to the study of HIE because of the multiple organizations, user types, hardware and software technologies, and sociopolitical motivations and goals involved in its composition. Though relatively few studies have examined an HIE network in operation, a sociotechnical approach was previously applied and shown to be appropriate to the study of HIE [5].

### Interorganizational Systems Implementations

An IOS is an IT-based system shared by two or more independent organizations [35]. Prior research on IOSs focused on the cross-organizational features of an STS [35,36]. While the implementation of IOS has been studied for decades across a wide array of industries, few studies have addressed health care, and fewer still have addressed HIE. IOS studies show the importance of: (1) learning from early adopters [37], and (2) evaluating the process of implementation to understand lessons learned and the real and perceived value of an IOS [38,39].

### From Interorganizational System to Health Information Exchange Implementations

Evaluations of information system collaboration require looking beyond a single focus and attending to multiple dimensions [40]. This perspective acknowledges that the collaboration of multiple stakeholders may hold the potential to create something new and better, as well as to create public value [41]. Similarly, a multidimensional perspective is required in evaluating the exchange of health information [42].

Evaluations of HIE and the benefits and challenges of exchanging health information have been studied in various contexts. For example, a 2011 study suggested that US $2 million in uncompensated care cost recovery is achievable with use of the nationwide HIE (now eHealth Exchange) as applied to disability determination [43]; and a more recent study estimated the resource utilization impacts resulting from using eHealth Exchange for emergency department visits [44]. Yet, few studies exist regarding the value of HIE at a statewide level. While these economic findings are important and may drive a sustainable IOS, understanding social value or motivation is important to HIE implementation.

As states end their cooperative agreements with the federal government, it is helpful to understand the challenges, successes, and lessons learned from an early on-boarder or implementation. While literature exists about information systems implementations across various industries, little is known about health systems implementations between public-private entities (eg, a private hospital and state HIE).

The aim of this case study is to provide insight into the technical, organizational, and governance aspects of a large private health system (Inova Health System) and the Virginia statewide HIE (ConnectVirginia EXCHANGE), organizations with the shared goal of exchanging health information. In this case study, the Blended Value Collaboration Enactment Framework, a multidimensional value framework [45], discussed later in this paper, provided a conceptual framework for evaluating the implementation process by which an organization becomes connected to a system to facilitate the exchange of information (ie, on-boarding), the first, to our knowledge, on-boarding to ConnectVirginia EXCHANGE.

## Methods

### Overview

The study design comprised direct observation, informal information gathering, document analysis, and semistructured interviews to study HIE implementation across technical, organizational, and governance dimensions. The study assessed the first, to our knowledge, on-boarding of a health care system onto the Virginia statewide HIE, ConnectVirginia EXCHANGE, using a formative evaluation of the implementation phase of the systems development life cycle. The study did not address the exchange of information, but rather the process of HIE implementation.

### Study Setting and Background

#### ConnectVirginia EXCHANGE

In March 2010, the Office of the National Coordinator for Health IT (ONC) awarded state cooperative agreements to the states and territories in the United States to develop infrastructure supporting the electronic exchange of health information. At that time, the Virginia Department of Health (VDH), the state-designated entity for Virginia, was awarded US $11.6 million. In September 2011, Community Health Alliance (CHA) was awarded a contract from VDH to build the Virginia Statewide HIE, ConnectVirginia. The organization to accomplish this statewide was subsequently initiated. Statewide HIEs were required to enable information exchange using standardized technologies, tools, and methods. Between September 2011 and February 2014, ConnectVirginia designed, tested, developed, and implemented three technical exchange services: (1) ConnectVirginia DIRECT Messaging (a secure messaging system), (2) ConnectVirginia EXCHANGE (the focus of this study), and (3) a Public Health Reporting Pathway (a pathway with VDH for public health reporting). ConnectVirginia EXCHANGE is a query/retrieve service in which a deliberate query passively returns one or more standardized continuity of care documents (CCDs; these provide a means of sharing standardized health data between organizations) from any other “system” on-boarded and connected to ConnectVirginia. This study examines and reports on the first implementation to ConnectVirginia EXCHANGE by Inova Health System (Inova).

#### Inova Health System

Inova [46] primarily serves the Northern Virginia and Washington, DC, markets and includes five hospitals with more than 1700 licensed beds and 16,000 employees. This comprehensive network of inpatient hospitals also includes outpatient services and facilities, primary and specialty care physician practices, and health and wellness initiatives. The inpatient facilities use a well known electronic health record (EHR), and the affiliated outpatient practices have access to that EHR. In keeping with Inova’s vision to increase value for patients and build an integrated network within and outside of their own hospitals, Inova became the first node to on-board to the ConnectVirginia EXCHANGE.

#### Electronic Health Record System

The EHR software involved in this study is primarily for mid-size and large medical groups, hospitals, and integrated health care organizations spanning clinical, access, and revenue functions. It provides an intranetwork data-sharing pathway (this specific EHR to this specific EHR), as well as an external data-sharing pathway (this specific EHR to a different EHR or system). Use of the external data-sharing pathway is the subject of this implementation study.

### Research Process

**Figure 1:**
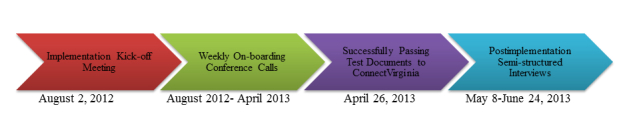
Evaluation timeline.

ConnectVirginia initiated and managed the on-boarding process. The on-boarding process for Inova began with a kick-off meeting on August 2, 2012, and concluded with a test to exchange electronic documents with ConnectVirginia on April 26, 2013 (184 total workdays). Along with Inova and ConnectVirginia, implementation involved two critical subcontractors: (1) MEDfx (the software vendor for ConnectVirginia), and (2) MedVirginia (the CCD content consultant). Figure 1 shows the evaluation timeline.

### Formative Evaluation of Information Systems Implementations

To assess the HIE implementation process from the perspective of an STS, this study used a formative evaluation methodology. Formative evaluations are widely used in young and developing initiatives to enable continuous improvement throughout the development and implementation stages [47,48]. From a practical perspective, this approach allows organizations to learn from past mistakes and develop better methods for assessing success [42,48]. This methodology allowed researchers to investigate the first implementation of ConnectVirginia EXCHANGE for a new and emergent type of system (ie, HIE) that is rapidly expanding across thousands of US health care systems.

To study IT implementations, Cooper and Zmud [49] proposed a diffusion process model of IT implementation that includes factors influencing implementation. Their model captures both the process and its context, subcategorized into stages. For example, their diffusion process model of IT implementation proposes six stages: (1) initiation, (2) adoption, (3) adaptation, (4) acceptance, (5) routinization, and (6) infusion. The present HIE evaluation covers only the *adoption* and *adaptation* stages of Cooper and Zmud’s model, which include: (1) gaining organizational backing for implementing IT applications, and (2) developing and installing IT applications, and developing and revising organizational policies and procedures for ongoing use of the IT applications. The classic system development life cycle recognizes four distinct implementation phases that can be used in IT evaluations: (1) preimplementation, (2) during implementation, (3) postimplementation, and (4) routine operation [42]. Evaluations such as this study conducted on the “during implementation” stage, use qualitative methodology [47,48]. It has been suggested that evaluations during this stage may be more important than those providing proof of outcomes [50], as the former can provide guidelines and lessons learned for others. The methods applied herein aim to extract valuable measures, and disseminate lessons learned for other HIE implementation efforts.

### Information Technology Implementation Measures

Evaluations of the exchange of health information can be challenging [51], partly due to the lack of any single model for HIE [50]. Implementation measures are generally chosen for their value to stakeholders [52]. Evaluations should determine not only how well a system works, but also how well it works for particular users in a particular setting [42].

Several measures have been applied to evaluations of IT implementations. Categories span different levels of abstraction including: (1) technical, (2) organizational, and (3) governance. In prior research, implementation measures pertaining to both technical and organizational dimensions included: (1) degree and type of data usage [50,53-55], (2) level of complexity of business processes [56], (3) completeness of information [47,50,54], (4) resistance to change [56], (5) unintended consequences [50,53], and (6) facilitators [47] and barriers [47,50] to implementation. Organizational and governance dimensions in implementations include: (1) communication [47], (2) trust [47], (3) organizational structure [53], (4) sustainability [12,54,57,58], (5) roles and power relations between participants [56], (6) levels of leadership commitment [47], and (7) representativeness and motivations of stakeholders [47,50,57].

The technical, organizational, and governance aspects of HIE, as well as their interactions with each other; provide a basis for the evaluation measurements currently utilized [42]. These measures were applied to the study of ConnectVirginia EXCHANGE within the context of a previously tested analytical framework for HIE [43].

### Analytical Framework

Enactment theory describes how people act within organizations [59]. When people carry out an act, they take into account their past experiences, events, and structures; determine a course of action; and then set that course into action. It is a form of social construction. Fountain’s technology enactment framework builds on enactment theory and considers that technical factors and organizational structures are embedded *within* each collaborating organization, and that the relationship between multiple factors is critical [60]. Others have suggested that while technical performance is a crucial element in any resulting information exchange between organizations, successful interorganizational data exchanges frequently hinge on organizational and governance factors [56,61-63]. However, other research notes that motivational factors and context can be the true underpinnings of collaboration [64,65]. As such, collaborations for information exchange require organizations to look beyond a single focus and give attention to multiple dimensions of collaboration [40].

**Figure 2:**
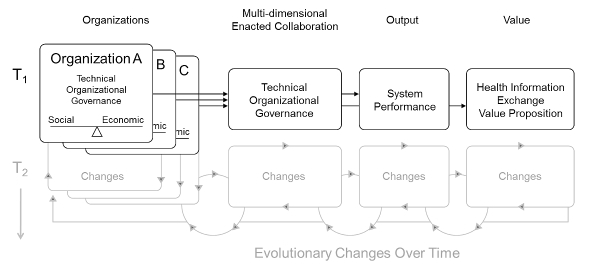
Blended Value Collaboration Enactment Framework [45]. T1 and T2= changes over time.

Based on the aforementioned work of Fountain, Schooley, and Emerson, and because of its prior use and demonstrated utility in assessing multi-organizational HIE efforts, the Blended Value Collaboration Enactment Framework was used to guide implementation and evaluation [45] (Figure 2 shows this framework). Framework development drew upon STS concepts and frameworks. Its importance for this study is that the framework: (1) considers each organizational stakeholder and its respective social and economic motivations for participating in HIE; (2) differentiates between technical, organizational, and governance dimensions; and (3) focuses on determining value propositions across stakeholders. For this study, and within the context of HIE, technical is defined as elements associated with the system or infrastructure; organizational is defined as elements associated with any and all of the stakeholders; and governance is defined as elements associated with decision making [45].

The above framework also considers the *value proposition* of HIE across stakeholders, including the social and economic motivations that lead to a more successful and sustainable HIE. A value proposition can be defined as the implicit promise of mutual value to the organization and its customers and/or partners [66]. For example, an in-depth case study of the fashion industry found that interorganizational value propositions could have both “hard” elements (economic gain, technological mastery, etc) and “soft” elements (brand identity, trust relationships, etc) [67]. Past research on HIE has illustrated that each stakeholder organization has its own value-driven motivations for participating in the exchange of health information, social including clinical (eg, “Is this the right thing to do for public health and wellness?”) versus economic (eg, “How does this impact our financial bottom line?”).

**Figure 3:**
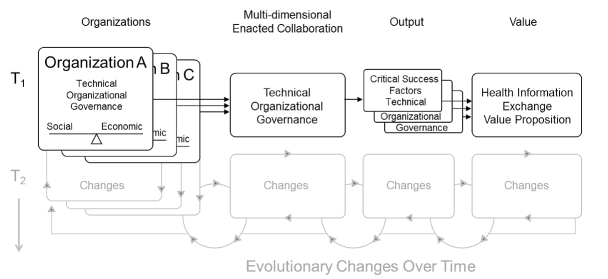
Blended Value Collaboration Enactment Framework. T1 and T2=changes over time.

The intended output of the Blended Value Collaboration Enactment Framework is the resulting system performance [45]. Since this study reports on the implementation of HIE and not on its actual use (from which system performance would be derived), the “output” section of Figure 2 has been modified in Figure 3 to reflect critical success factors, as is more appropriate for implementation studies [68]. The framework also proposes that motivations and value propositions may change of over time (T_1_ and T_2_ in Figure 2). This study investigates only the implementation stage and does not evaluate how these dimensions change over time. Therefore, only the unshaded areas of the framework are germane to this analysis.

### Observation, Informal Information Gathering, and Document Analysis

Data collection took place August 2012-June 2013 by one of the authors, who was the external evaluator to ConnectVirginia and not part of the implementation team. A total of 36 hours of observation of the on-boarding process occurred across planning, coordination, implementation, and problem-solving meetings held either in-person or via conference calls. Each organization was represented in each meeting, and the meetings provided an environment for conducting informal information gathering. Various documents, such as meeting notes and detailed meeting minutes, were also analyzed.

### Semistructured Interviews

Qualitative methods were employed to understand *how* and *why* the factors in each dimension contribute to or influence the overall implementation and value derived. At the end of the project (between May 8 and June 24, 2013), 12 60-minute, semistructured in-person interviews were conducted across the five participating organizations (ConnectVirginia, MEDfx, MedVirginia, EHR vendor, and Inova). Table 1 provides additional details about the interviewees. Purposive sampling was used to select interviewees based on: (1) their belonging to one of the above-mentioned organizations, and (2) their degree of participation in the on-boarding process. For example, individuals who participated in the majority of project manager (PM), technical, or system testing meetings were invited for interviews, and all invitees agreed to participate. Persons such as consumers of the exchanged health information (ie, clinicians) were not interviewed because, at the time of the study, there was no routine exchange of information for real-world use. Interviews were conducted by one of the authors with expertise in conducting interviews, and who was also the external evaluator to ConnectVirginia and not part of the implementation team.

Interview questions were designed to develop a clearer picture of the on-boarding process, to recreate the actual timeline, and to support information from calls, documents, and informal information gathering. Table 2 provides a sampling of interview questions. Not all questions were appropriate for all interviewees, and therefore were not asked to all interviewees. Likewise, if interviewees had in-depth knowledge of a particular process, secondary questions were asked that may or may not have been used for other interviewees.

**Table 1:** Interviewee’s by organization, position, and role.

Organization	Position	Role during implementation
ConnectVirginia	Executive Director	Oversight
	PM	Daily operations management of the implementation
MEDfx	Chief Operations Officer	Oversight
	PM	Daily operations management of the implementation
MedVirginia	Chief Information Officer	Oversight
	Systems Analyst	CCD content validation
EHR vendor	Application Support Specialist (x3)	Provided vendor support during the implementation
Inova	Executive Vice President and Chief Technology Officer	Oversight and internal champion
	Senior Vice President and Chief Information Officer	Oversight
	PM	Daily operations management of the implementation

**Table 2:** Sample interview questions.

Sample interview question types	
**Technical**	
	What were the initial technical processes involved in on-boarding to ConnectVirginia?
	What technical advances and/or information could have streamlined the on-boarding process?
	What technical challenges emerged and how were they addressed?
	Were any technical “workarounds” employed? If so, please explain.
	What technical processes were particularly successful and why?
	To what extent was the technical assistance that you received helpful?
	Please describe your current level of HIE (eg, within your organization, outside your organization, labs, etc).
**Organizational**	
	To what extent did organizational leadership impact the on-boarding process?
	What is the value proposition of on-boarding to ConnectVirginia?
	What organizational challenges emerged and how were they addressed?
	What is needed to have HIE become a standard of care?
	What was particularly successful regarding organizational leadership?
**Governance**	
	What were the key elements of the governance structure within your organization for on-boarding to ConnectVirginia?
	What governance structures do you see as vital for sustainability or growth of HIE across ConnectVirginia?
	To what extent were on-boarding guides governing implementation useful, helpful, or challenging?

### Data Analysis

**Figure 4:**
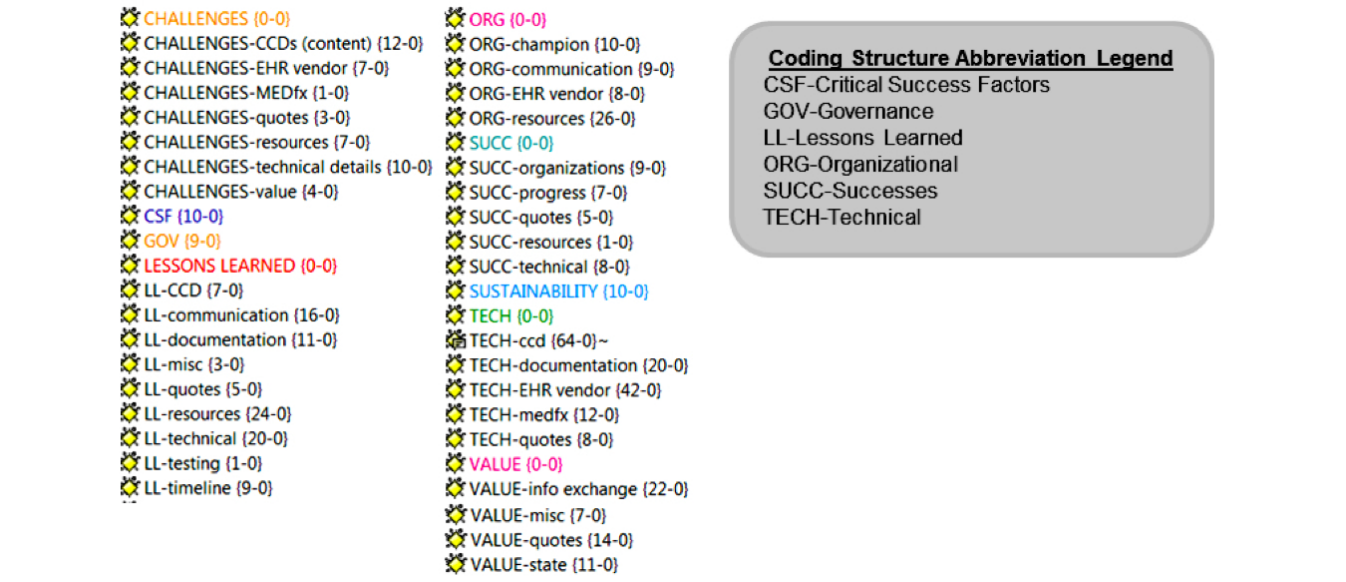
Coding structure. CCD=continuity of care document, EHR=electronic health record.

Interviews were transcribed verbatim and imported into ATLAS.ti, a qualitative data analysis software application [69]. The Blended Value Collaboration Enactment Framework was used to guide the analysis. Each dimension from the framework (technical, organizational, and governance) was used to provide a predefined coding structure frame. The framework also provided predefined coding categories for critical success factors and value proposition of the implementation (Figure 4 shows this coding structure). Interview transcripts were coded and data attributed to the appropriate category. Challenges, lessons learned, and sustainability were not part of the predefined categories; so new code categories were created.  Data were coded and then checked by multiple researchers for interrater reliability.

All interview data were analyzed to understand the challenges, successes, and lessons learned within each dimension (technical, organizational, and governance) and within each collaborating organization (ConnectVirginia, MEDfx, EHR vendor, and Inova) of on-boarding to an HIE network (in this case ConnectVirginia EXCHANGE). Data were also analyzed to gain insight into issues that would provide meaningful information regarding factors contributing to success, value, and sustainability. Using ATLAS.ti, this was performed by comparing organizations and code families. For example, all stakeholder groups and all codes related to challenges were selected to compare stakeholder positions relative to challenges. Data were then exported to Excel to view frequencies.

## Results

### Lessons Learned

The lessons learned, as derived from the challenges and successes, are summarized in Table 3 across technical, organizational, and governance dimensions. Each subsequent subsection (technical, organizational, and governance) serves to unpack those lessons learned in terms of challenges and successes. Collectively, the findings not only provide a retrospective account of Inova’s efforts in on-boarding to ConnectVirginia EXCHANGE, but also offer insights into various other stakeholders for future on-boarding efforts.

**Table 3:** Lessons learned from challenges and successes by dimension (technical, organizational, and governance).

Lessons learned	Challenges	Successes
Technical	Determine the most efficient environment for testing, decoupled from decision processes, actions, and dependencies from other stakeholders.	Willingness to develop workarounds to unexpected software challenges, such as incompatible EHR versions.
	Provide oversight and follow-up to increase technical understanding of appropriate on-boarding guides across all stakeholders.	Gain commitment from implementation site to set high priority on HIE implementation.
	Use an EHR system specific CCD for validation, not a vendor supplied CCD template.	
	Conduct testing and implementation in clearly communicated iterations.	
	Articulate goals and priorities with vendors.	
	Understand roles and required resources in order to minimize time gaps and maximize efficiency.	
Organizational	Account for competing IT priorities across organizations.	Participation of health system leadership.
	Understand, communicate, and appreciate varying stakeholder value proposition/motivations.	Timely and accurate communication, especially by and between the HIE and the health system.
		Allocate appropriate human resources at the outset.
Governance	Ensure governance is in place, including policies, procedures, guidelines, and oversight across all organizations.	Project champion possesses decision-making power, or, as needed delegates appropriate decision making power to others.
	Obtain commitments from governance body early on to facilitate project continuity.	

### Technical Dimension

This on-boarding effort between Inova and ConnectVirginia entailed a range of technical activities and coordination to achieve success. There were four major challenges: (1) the testing environment, (2) the on-boarding guides, (3) the CCD, and (4) the vendors, and various successes contributed to the lessons learned.

### Challenges

#### The Testing Environment

Regarding the testing environment, many interviewees felt that significant time early in implementation was spent determining which Inova environment would be used for testing. Inova had two environments capable of producing CCDs: (1) test, and (2) proof of concept (POC); the latter is connected to the EHR vendor’s connected network. Inova elected to use the POC environment, which created work inefficiencies across Inova and the EHR vendor. Analysis revealed the challenge, every time Inova wanted to conduct software tests, it required the EHR vendor team to sign into POC, input scenarios, and start the testing. The EHR vendor team considered these manual steps as wasting time, which resulted in testing delays. An interviewee discussed this issue,

Had we chosen in the very beginning to use the test, we would not have had any difficulty setting up test patients to test in ConnectVirginia...[New on-boarders] should think long and hard about their testing environment and it should be done early in the process.

An interviewee

A review of meeting notes shows that this process took approximately four weeks, whereas participants believed it should have taken less than two weeks. During this time, frustrations from Inova, ConnectVirginia, and MEDfx were observed by one of the researchers, who was consistently on the conference calls. These frustrations were a direct result of Inova’s reliance on the vendor related to CCD testing.

The lesson learned from the challenge of choosing a testing environment was that this should be done independent of other stakeholder actions. Determine the most efficient environment for testing, decoupled from decision processes, actions, and other dependencies from other stakeholders. 

#### The On-Boarding Guides

ConnectVirginia provides four guides to assist on-boarders: (1) checklist, (2) implementation, (3) testing, and (4) content, which, unless specified, will be referred to collectively as “on-boarding guides.” Not all guides are meant to be read by all parties. For example, the EHR vendor should read content guides, and testing guides should be read by the on-boarding organization. Many of those involved with this implementation had the perspective that the on-boarding guides were not read by the correct parties (or at all by anyone), but that, had they been, certain on-boarding tasks could have gone more smoothly and challenges could have been avoided. For example, the *checklist* is to assist in creating a shared understanding of the data needed, relative to the data available; for example, it requires data on all the laboratory tests that could possibly be run by the on-boarding organization. Several interviewees suggested that this document is long, complex, difficult to read, and overwhelming to the reader, and therefore does not get completed. However, it is an important element in setting up the CCD for validation. An interviewee suggested that it would be better to go through the checklist section by section to determine whether the data are present or absent.

The *implementation* and *testing* guides are provided to create a shared understanding and level set of expectations about the requirements for implementation and testing. From the meeting notes and interviews, it was clear that these guides were not read to the degree needed for the project. An interviewee explained, “Regardless of how painful [you] think it might be, read the [on-boarding] guides cover to cover.” This same interviewee thought the on-boarding guides were very well written and provided plenty of the necessary helpful information. Another interviewee suggested that discussing the on-boarding guides among the implementation group would allow time for conversation and might raise issues not easily discovered by an individual, which are illuminated when discussed in a group. Last, the *content* guide is provided to streamline CCD validation testing. This guide defines what the CCD should have in terms of object identifiers and the corresponding descriptions. Several interviewees felt many misunderstandings could have been avoided had this guide been read and discussed.

The lesson learned was to provide oversight and follow-up to increase technical presentation, reading and understanding of appropriate on-boarding guides across all stakeholders.

#### Continuity of Care Documents

The challenges with CCDs and HIEs are well documented in the literature [43,45,70]. Initially, the EHR vendor wanted to provide only template CCDs and not CCDs specific to Inova’s EHR system, which includes customization. MedVirginia, which conducted the CCD content validation, requested CCDs specific to Inova’s system to ensure that a CCD could be sent and received from Inova’s customized and nuanced system. Using a standardized vendor template does not account for health system customizations and risks not passing validation in the eHealth Exchange environment. An interviewee noted,

A scrubbed [vendor template] CCD will probably not have any issues, but when it comes to testing a CCD from the actual system, there are going to be issues, so you should not expect that just because the sample [vendor template] passed, that the node CCD will pass; it probably won’t.

An interviewee

Meeting notes and interviews reflect the opinion that this issue required too much time devoted in isolation and should have been handled along with other on-boarding tasks. For example, many participants commented that the technical piece (getting systems to talk) and the content piece (the CCD) should have been conducted in parallel, rather than sequentially. An interviewee thought the CCD issue could have been managed in parallel to solving an issue with handshakes (ie, bidirectional system-to-system acknowledgement),

While the CCD is the end point, the handshake had issues. [The Inova] server has to be recognized by MEDfx before any exchange can even happen.

An interviewee

Once CCD validation was underway, many felt that the on-boarding process, while excellent and thorough, needed to strike a better balance between the acceptable and the desirable. Several interviewees felt that if all parties had agreed on prioritization of issues (ie, with less attention to details that do not matter), the CCDs could have passed testing much sooner and had an earlier completion date.

#### The Vendors

For example, the top priority for the EHR vendor was to get the technical pieces to work from their end of the HIE. The vendor’s goal was to focus on passing a CCD; not passing a CCD that conforms to the updated eHealth Exchange specifications. Since EHRs undergo a wide variety of customization, this is a critical requirement nuance relative to future participation in eHealth Exchange. While it may have been *acceptable* to use a template CCD, it was *desirable* (because of customizations and future eHealth Exchange participation) to conduct CCD validation with a system CCD. Participants other than the EHR vendor felt the vendor was not focused on developing a technology to support *all* other stakeholder value propositions and motivations.

An interviewee described how differing priorities across stakeholders impacted the HIE outcome,

Given the experience with [the EHR vendor], it is important to communicate to clients that this [their decision to limit the CCD] might impact what type of approval is granted at the end of on-boarding, because [Inova] ended up with a conditional approval based on some of the things that we knew [the EHR vendor] wasn’t going to budge on.

An interview

As noted above, Inova received only conditional approval as an HIE participant/node. The conditional approval was the result of a 90 day medical record date range limitation imposed by the EHR vendor’s CCD implementation for this project. The US Social Security Administration requires *more* than three months of medical records in order to conduct disability determinations, but the vendor’s implementation allowed only three months’ worth of data. In this regard, an interviewee observed,

Once we got into the testing process, that unveiled a lot of proprietary issues with [the EHR vendor], even though they say their CCD is compliant. They have certain things that are built into their product, mainly from a competitive stand point. Inova was very reliant on [the EHR vendor], and I think somewhat unaware where those proprietary issues might impact on-boarding to any statewide HIE.

An interviewee

Interviewees had comments about both the EHR vendor and MEDfx. Many considered the EHR vendor inflexible and unknowledgeable, especially with regard to the 90 day medical record date range limitation and requirements by the U.S. Social Security Administration, a critical component in the value proposition. Interviewees also felt that MEDfx was developing as the project advanced. According to interviewees, this project represented the first implementation of the latest eHealth Exchange compliant gateway. MEDfx, the gateway provider, had completed development of the gateway in June 2012, but had not yet completed testing. Thus, Inova became the test bed for the gateway. This process of testing during implementation contributed to the perception that MEDfx was developing as the project was advancing. Both vendors (the EHR vendor and MEDfx) had to fix some bugs that were exposed during testing, and some interviewees thought the fixes should have been completed before implementation or at least done more expeditiously.

The lesson learned was to use an EHR system specific CCD for validation, not a vendor supplied CCD template.

Data gathered from weekly on-boarding meetings, together with document review, reflected the EHR vendor’s resistance to meet the needs of a maturing and broader HIE, such as a statewide HIE. An interviewee noted,

[The EHR vendor] is a very restricted vendor around allowing third parties to do things like this. We probably lost a month in this go around.

An Interviewee

Another interviewee said, “I regret that anyone thought [the EHR vendor] would change their mind.”

The lesson learned was that given the complexity of managing expectations across multiple stakeholders, conduct testing and implementation in clearly communicated iterations.

Several other challenges surfaced regarding software versions that were incompatible with the latest data exchange standards. The EHR vendor software version that Inova used for this implementation (released in 2010) was not compliant with the upcoming eHealth Exchange specifications. The vendor will not release a version compliant with the new specifications until the 2012 version. Discussions with the EHR vendor suggested that, due to the relative newness of the current 2010 version, health systems will likely not deploy the 2012 version for some time. This discrepancy in EHR specifications created significant challenges, in terms of the CCD content, to completing on-boarding. However, the EHR vendor’s perspective differed from that of ConnectVirginia regarding the ability to on-board, saying, “We have many other clients that have on-boarded to eHealth Exchange, and none of them have these [CCD content] issues that ConnectVirginia is citing.” In response, ConnectVirginia participants explained that the clients to which the EHR vendor refers had been on-boarded under the old Nationwide Health Information Network specifications established by ONC, rather than the new and required eHealth Exchange standards established in September 2012. ConnectVirginia must on-board to eHealth Exchange under the updated eHealth Exchange specifications. Thus, misunderstandings about the required specifications caused significant implementation delays. Because this was the first on-boarding with the EHR vendor, there were many unknowns. It became apparent that an early meeting with the EHR vendor was critical. Many felt this would have created a shared understanding of some of the nuances of each other’s systems and of stakeholders’ motivations, while also fostering conversations about how each system adheres to the implementation specifications.

The lesson learned was to establish early meetings with vendors to articulate goals and priorities.

Interviews revealed challenges with understanding each stakeholder’s roles. Several interviewees felt that time was lost determining who was responsible for certain things, and tasks were not done because one person thought another was responsible. Likewise, better understanding was needed of the technical resources available: (1) Are the right people working in the right place?; and (2) Is the testing environment one that will facilitate on-demand testing?. An interviewee felt that two MEDfx people had the knowledge collectively, but lacked depth individually. This type of situation led to delays or multiple attempts to get questions answered. Another interviewee felt it was critical to have representation across integrated delivery teams, primarily because policy issues needed to be addressed saying, “An interface group will build a pipe for your data to pass, but there are lots of rules regarding audit streams.” A majority of interviewees thought many of these late questions or realizations could have been avoided by earlier and better understanding of the on-boarding guides provided to Inova and the EHR vendor. Much that was done was conducted sequentially; many interviewees thought the technical piece (getting the systems to talk) and the content piece (the CCD) should have been done in parallel and speculated that doing so would have saved a lot of time.

The lesson learned was to conduct clear communication early on to discuss and understand roles and required resources in order to minimize time gaps and maximize efficiency.

### Successful Software Redevelopment

Regarding the incompatible software versions described above with the 2010 EHR version, almost all the interviewees with knowledge of this issue commented on MEDfx’s willingness to develop new code to address this challenge. While software redevelopment took time, causing unanticipated delays, everyone saw this as a significant success. A participant summarized the thoughts of many,

We put a lot of responsibility on MEDfx to make adjustments on their side to accommodate the fact that [Inova] was running a version that only supported 2010. Thankfully, they were flexible enough to accommodate that.

A participant

While Inova could have on-boarded to ConnectVirginia without this modification, ConnectVirginia would not have been able to on-board to eHealth Exchange, thus minimizing the value for Inova and any organization on-boarding after Inova. All interviewees felt that getting Inova on-boarded was a great success in and of itself.

All stakeholders had competing priorities, but participants noted that Inova’s may been the most significant. Although they were in the middle of an EHR implementation, they chose to pioneer on-boarding to the ConnectVirginia EXCHANGE.

The lesson learned was to gain commitment from technology stakeholders to be willing to develop workarounds to unexpected software challenges, such as incompatible EHR versions.

Another lesson learned was to determine conflicting priorities across stakeholders at the outset. Gain commitment from implementation site to set high priority on HIE implementation.

### Organizational Dimension

Organizational factors were also instrumental to the success of the Inova on-boarding experience. Concurrent EHR implementation and strong leadership contributed to the organizational challenges, successes, and lessons learned.

### Challenging Competing Priorities

The major challenge involved a concurrent EHR implementation at Inova and understanding each stakeholder’s value proposition and motivation. Early on, the concurrent EHR implementation created a situation of competing priorities. However, once roles were more clearly defined regarding the ConnectVirginia implementation, it was felt that resources were available and engaged. As one interviewee observed, “[The Inova internal champion] kept us [Inova team] moving because we were very busy with a lot of other stuff including [EHR] implementation.”

The lesson learned was to account for major competing IT priorities at each participating organization. A concurrent EHR implementation will likely compete directly with the HIE implementation.

The value proposition and corresponding motivation for on-boarding to ConnectVirginia EXCHANGE varied with the stakeholder. Several Inova participants commented that, although the initial economic value proposition to Inova was nonexistent, the motivation to move forward was very well aligned with their vision to “reinvent hospital-based care to increase value for our patients” and to “look outside our hospitals to build an integrated network of providers and programs to support our community.” The culture of this vision was embedded in Inova employee beliefs. The words of several were summed up by one Inova interviewee, “On-boarding to ConnectVirginia [exchange] aligns with the Inova vision, fulfills our desire to be part of transforming the Commonwealth [of Virginia] into a great place to be a patient, and success for Inova means great benefit for the community.”

Additional motivations for Inova involved the desire to be leaders in the HIE trend. Some interviewees questioned whether or not the EHR vendor understood why this was so important to Inova and ConnectVirginia independently and collectively, and interviewees suggested the EHR vendor was sometimes argumentative with requests from the on-boarding team, “Their [the EHR vendor’s] resistance to ConnectVirginia’s success is what concerns me.” To the other stakeholders, it seemed that the EHR vendor did not have a clear motivation and was simply responding reluctantly to client requests. These differing views on value created communication breakdowns, frustrations, and inefficiencies in the on-boarding process. These breakdowns and frustrations were observed numerous times on various conference calls with the implementation team. There were times when it would take three or four calls to resolve one issue. Such events led to inefficiencies in the on-boarding process.

The lesson learned was to understand, communicate, and appreciate varying stakeholder value proposition/motivations.

### Successful Leadership

Leadership was an important factor in this on-boarding process. Almost all interviewees commented that Inova’s internal champion, the Executive Vice President and Chief Technology Officer of Inova, was a critical component in the success of the project. Many suggested that his role on the ConnectVirginia Governing Body put him in a position to be an internal champion not only for Inova, but for ConnectVirginia as well, with his solid understanding of what ConnectVirginia was trying to accomplish and why it was important. During an interview, he stated,

I thought Inova needed to learn what HIE is and needed to get its feet wet with the on-boarding so it could be connected. I believe in HIE.

Executive Vice President and Chief Technology Officer of Inova

Scheduling sensitivities around Inova’s EHR implementation created some time periods when key people were unavailable. When this resulted in a lack of progress between meetings, the Executive Vice President and Chief Technology Officer of Inova could help guide the Inova team with managing those competing priorities. His unique combination of being an internal champion and a decision-maker greatly enhanced the success of this project. An interviewee further qualified the role of an internal champion, “This cannot be a technical champion, but a true champion...a true leader.” Leadership in terms of project management was considered solid. Several interviewees commented on Inova’s PM, and one summarized the words of many,

She [the PM] was prepared, answered emails promptly and completely, and executed well. It was extremely helpful to have her.

Interviewee

The lesson learned was that the participation of health system leadership is critical to success.

Communication was another area of success, and many felt that PMs from both Inova and ConnectVirginia greatly contributed to that success. As mentioned above, the PM from Inova was always prepared and answered emails promptly, and many others commented on the PM from ConnectVirginia. An interviewee capsulized ConnectVirginia’s communication efforts,

I appreciate the fact that they controlled a lot of the documentation. They scheduled the weekly calls, set up the agendas, sent out the minutes, and managed any outstanding items across everyone.

Interviewee

Several interviewees mentioned that the meeting minutes were very thorough.

The lesson learned was that timely and accurate communication, especially by and between the HIE and health system, is essential.

Despite the fact that this project competed with resources for the Inova EHR implementation, many felt there were appropriate resources. However, most interviewees observed that initially the correct resources were not allocated. Since this was the first on-boarding, there were vague expectations about the level of and appropriateness of resources. Teams required skills sets, knowledge, and experience that were not available at the outset. An interviewee noted,

Too many assumptions were made. We need to have a better kick-off to level set expectations and roles.

An Interviewee

Most interviewees felt that, by the second month, the teams were appropriately resourced, and what was originally a challenge became a success. Several commented that there was not a lot of movement regarding the resources, which added to the teams’ strength individually and collectively. Another interviewee summed up the lesson learned,

Put your best resources around standing this up, because it requires you to pay attention to detail. This is more than an IT project; this is not a simple interface project.

An Interviewee

The lesson learned was to allocate appropriate human resources at the outset.

### Governance Dimension

Intra and interorganizational decision-making power and clear role definition have been shown to decrease intra and interorganizational issues [71]. Furthermore, the nature of the relationships between decision makers is important in navigating variable governance processes and structures and in sharing decisions. Governance is the establishment of oversight, standardized policies and procedures, and mechanisms to ensure operation of an organization [72]. Thus, governance factors such as the on-boarding guides, project resources, and a project champion contributed to the lessons learned and the critical success factors of the Inova on-boarding project.

### Challenging Identification of Appropriate Policies and Guidelines

Identifying the appropriate policies and guidelines across stakeholders was challenging, as was selecting the best people to provide oversight. Unfortunately, a structured governance process did not predate this project, this was the first time this particular group of organizations had worked together, and the first time on-boarding to ConnectVirginia had taken place. As noted previously, providing oversight and policy enforcement for people to read the on-boarding guides proved challenging. If on-boarding guides had been read thoroughly, it may have been easier to identify the correct governance resources or, at least, ask questions regarding resource selection. Regarding the governance structure for the project, one interviewee commented, “The technology supports the business, but the business does not go anywhere without the right folks.” Another interviewee said that she was, “...challenged to put together a governance group that could attend the weekly on-boarding meetings, as those were a great way of getting decisions made.”

These deficits resulted in the organizational and technical challenges described earlier, including missing nuances of the on-boarding process, difficulties selecting key project resources at the outset of the project, and not providing a structure whereby team members could request guidance in resource selection. Fortunately, these issues were identified and quickly rectified within the first two months of the project.

The lesson learned was to ensure governance is in place, including policies, guidelines, and oversight across all organizations.

Most interviewees agreed that the on-boarding guides provided by ConnectVirginia were useful in explaining appropriate governance such as policies, procedures, etc, once they were read. The challenge was getting people to read them. Time was short, priorities competitive, and resources thin. However, several interviewees agreed that thorough reading of the on-boarding guides just before the kick-off meeting would have helped ensure that proper governance decisions were made, especially in regards to policy decisions. It was also thought that a thorough reading would have mitigated some downstream misunderstandings and poor understanding of the system requirements. Other than that, some interviewees thought the ConnectVirginia PM could have done more to ensure that those responsible for governance understood the on-boarding guides pertaining to their part of the project. For example, at one on-boarding meeting, one individual from Inova asked to go through one of the on-boarding guides. An interviewee commented, “Sometimes we all need to have our hand held, and if that is what it would have taken to make sure everyone went through the on-boarding guides, then so be it.”

The lesson learned was to obtain commitments from the governance body early on to facilitate project continuity.

### Successful Project Champion

Most interviewees agreed that the real success in this project, from a governance perspective, was having a project champion with decision-making power. Several times policy or procedure decisions needed to be made; and because the Executive Vice President and Chief Technology Officer of Inova was involved, the appropriate questions were asked and decisions made. An interviewee gave a good example of how the project champion provided appropriate decision-making authority,

When something comes up as an issue, helping to figure out if it is a technical issue or a policy issue. Then figuring out whose issue it is; is it a node [hospital system] issue, is it the vendor, or is it ConnectVirginia?

An Interviewee

In these situations, the Executive Vice President and Chief Technology Officer of Inova was able to provide guidance on issues involving the node or Inova’s EHR vendor. Regarding MEDfx and issues attributed to them, the PM had authority to provide the guidance needed to move forward. Regarding CCD content validation, which was conducted by MedVirginia, it was felt that the person on the on-boarding calls did not have decision-making authority, and thus needed to seek guidance after the call. Yet, interviewees felt her follow-up communications were timely and comprehensive.

The lesson learned was that a project champion is essential who possesses decision-making power, or, as needed, delegates appropriate decision-making power to others.

It was clear to participants how critical it was to ensure from the beginning that a proper oversight structure is in place, including the people involved in the project. As mentioned in the challenges section, the on-boarding guides provided by ConnectVirginia helped to provide guidance in this regard and to identify a governance structure. In addition to identifying project resources, early identification of an internal champion is essential for project success. But, as one interviewee mentioned, it is sometimes difficult to have the internal champion with decision-making authority at the weekly meetings.

### Stakeholder Perceived Value

Analysis of interviews, observations, and project documents, taken together, also revealed a crosscutting theme in terms of the goals, priorities, motivations, and perceived value of engaging in HIE. Earlier on in the implementation, an important success factor would have been to have a better understanding of how well the organizational goals of each participating organization aligned with one another; the implementation priorities and motivations (social and economic) for each organization to participate; and the perceived value that each organization expected to gain as a result of participating. These are illustrated in Table 4. Providing this information may have avoided some of the challenges, constraints, and tensions experienced, especially with the EHR vendor.

It was felt by many interviewees that had something like the below matrix existed, that clarity and insight would have been gained early on.

**Table 4:** Matrix of goals, priorities, motivations, and perceived value propositions across implementation stakeholders.

	ConnectVirginia	Inova	MEDfx	EHR vendor
Implementation goal alignment	Aligned	Aligned	Aligned	Not aligned
Implementation priority	High	High	High	Low
Motivation	Social high	Social high	Social low	Social low
	Economic moderate	Economic low	Economic high	Economic low
Perceived value	Provides exchange of medical information	HIE leader in the state medical information at the point of care	Fulfills the contract terms	None apparent
	Fulfills the contract terms	Social Security Administration disability determination		

## Discussion

### Principal Findings

**Figure 5:**
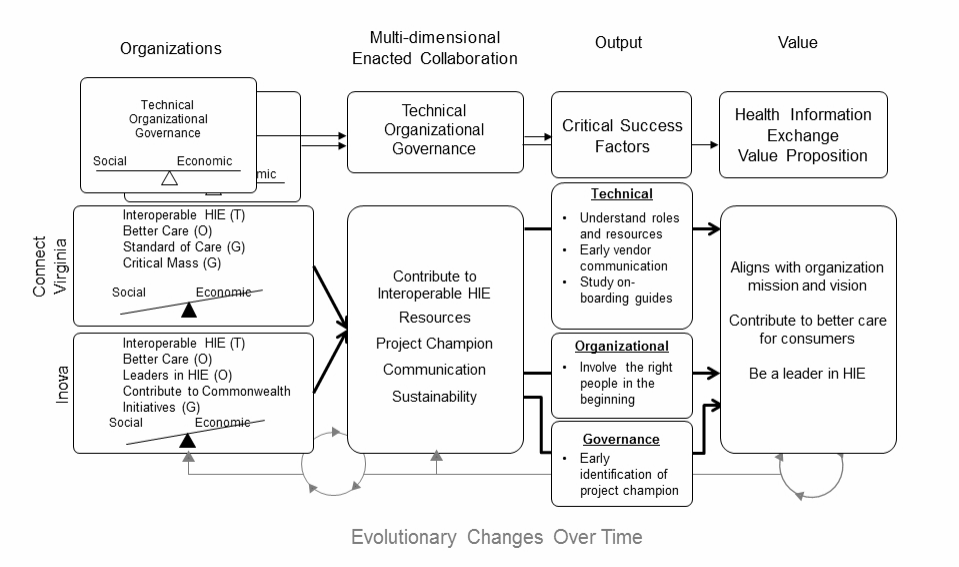
Blended Value Collaboration Enactment Framework as applied to findings. T=technical, O=organizational, G=governance, and HIE=health information exchange.

The main findings of this case study included several challenges, successes, and lessons learned during the implementation of a health system on-boarding to a statewide HIE. Figure 5 shows a summary of the Blended Value Collaboration Enactment Framework as applied to these findings and illustrates the technical, organizational, and governance collaboration that took place between ConnectVirginia and Inova, along with critical success factors and associated value proposition details.

This case study demonstrates that *interorganizational governance* of HIE implementation is replete with interrelated and overlapping technical, organizational, and governance issues. The complexities of collaboration appear to assist as well as detract from realizing a set of common goals. For the expanded view of HIE (ie, across states and the nation), the broader significance of this case study is the proposition that successful implementation of a large-scale emergent HIE system should consider the expected and realized blended value across all participants. Consistent with the literature, while economic value is an important goal, the organizations presented here have regarded the value proposition primarily to include social value, believing that economic value will follow at some point in time.

As mentioned earlier, the sociotechnical approach allows for understanding independent linkages between complex social and technological components. Much of what was learned from this first on-boarding effort is related to accomplishing tasks earlier in the process, rather than allowing them to be discovered in course. While this may be common knowledge in more established IT implementations, the field of statewide HIE on-boarding implementations is undeveloped in this area. The interviews illuminated some common issues such as interoperable HIE and better care. However, it was notable that both organizations, when asked about their motivation to collaborate, cited the social good that would result from creating a critical mass and contributing to the Commonwealth’s HIE initiative. Since Inova has no other organizations with which to exchange, and ConnectVirginia does not yet receive revenue from on-boarding organizations, both organizations were motivated primarily for the social good.

### Application of the Framework

Turning to the Blended Value Collaboration Framework as applied to the findings, Figure 5 illustrates the interdependent linkages between the technical, organizational, and governance dimensions for each organization. When these individual dimensions come together in the collaboration there are contributing factors that facilitate enactment of the collaboration. At this early point in the project, both organizations were heavily weighted toward social motivations, recognizing that as ConnectVirginia matures the economic motivation will grow more prominent.

Frequently in public-private collaborations, the value propositions of collaborating organizations are not aligned. However, in this case, we found the value propositions between ConnectVirginia and Inova to be very well aligned and centered on organizational missions and goals, better care for consumers, and being leaders in HIE. This is very consistent with the IOS literature [41,42]. As mentioned earlier, this study focused on the implementation, so there was no usage, and only one period of time was studied. Thus, the evolutionary changes over time portion of the framework were not addressed in these findings (gray portion in Figure 5). This sociotechnical approach facilitates the consideration of the social and the technical perspectives, and their contribution to the overall value proposition.

Supporting Mody’s [32] assertion, describer earlier in this paper, that social considerations could exert more pressure than economic considerations, this framework highlights social reasons as very significant motivators for early adopters/implementers, frequently outweighing economic motivations. Lacking a framework that considers social motivations, the natural tendency in IT projects might be to analyze, or only consider, economic motivators, and then judge the value proposition on that basis. The Blended Value Collaboration Enactment Framework fills a gap in, and makes a contribution to, the STS framework literature. Its application promotes the assessment of a wide range of observed issues (across technical, organizational, and governance dimensions) in relation to an interorganizational health IT implementation, comparing them with IOS goals, motivations, and intraorganizational priorities, and then determining the success factors and value propositions from the results. Used as a heuristic, the framework may provide for a broader and more inclusive evaluation of an IOS health IT implementation. Furthermore, the current framework enables examination of changing motivations and value propositions over time.

Future studies should revisit the findings reported here to analyze such changes. As time progresses and ConnectVirginia matures, an increased economic motivation is expected from both organizations. In the future, organizations are also expected to on-board primarily for perceived potential economic factors, although these are yet to be realized. Future research should assess a wide range of economic and clinical factors associated with HIE value; while continuing to define, include, and broaden social factors and public value. Mixed-method case studies can be used in this regard to more fully understand the breadth and depth of mediating, moderating, and control variables to assess in future quantitative studies. Studying HIE implementations broadly across the United States through survey research is desperately needed as most studies, including those referenced in this manuscript, consist of small sample sizes. In terms of content, the value proposition in HIE is a moving target, as both the act of HIE and entity called HIE continues to evolve and change. New government requirements and incentives, new business models to facilitate HIE, and increased societal demand for better and less expensive health care are expected to continue to shape the HIE landscape. Knowing this evolution will occur should not deter near-term research. These studies are needed in order to make the ongoing practical impact discussed at the outset of this manuscript, to address the triple aim of health care broadly across regions.

### Limitations

This case study examines the implementation of one health system on-boarding to a statewide HIE. As such, generalizability may be limited. Another factor holding potential to contribute to this limitation includes that the Chief Medical Information Office of the health system was the statewide HIE Governing Body Vice-Chair, which may have served to introduce motivations and bias that a different implementation would not have experienced. However, it may also be commonplace for existing health care leaders in regions and states to take a strong role in HIE governance. Further research is needed to apply the principles from this study to other implementations, so as to gain generalizability of the findings.

### Conclusions

This study focuses on the evaluation of HIE implementation in Virginia. From a practical perspective, the study provides a set of lessons learned for others who are implementing systems across a statewide HIE. This study also includes considerations for eHealth Exchange implementation. As mentioned earlier and substantiated in the literature, on-boarding to eHealth Exchange is part of the economic value proposition equation. On-boarding to ConnectVirginia with a CCD that will not pass testing when ConnectVirginia on-boards to eHealth Exchange eliminates a critical value proposition component. From a methodological perspective, it provides an example of how such an HIE implementation can be studied, and from a theoretical perspective, this study builds on the literature on IOS for health care, addressing the core questions: (1) What value propositions motivate an organization to participate in HIE implementation?; and (2) What success factors should be targeted in HIE implementation evaluation?.

## Abbreviations

CCD: continuity of care document
CHA: Community Health Alliance
EHR: electronic health record
HIE: health information exchange
Inova: Inova Health System
IOS: interorganizational system
IT: information technology
ONC: Office of the National Coordinator for Health Information Technology
PM: project manager
POC: proof of concept
STS: sociotechnical systems
VDH: Virginia Department of Health
